# Glutamatergic dysfunction, neuroplasticity, and redox status in patients with functional movement disorders

**DOI:** 10.1192/j.eurpsy.2023.2158

**Published:** 2023-07-19

**Authors:** B. Demartini, V. Nisticò, C. Benayoun, A. C. Cigognini, R. Ferrucci, A. Vezzoli, C. Della Noce, O. Gambini, A. Priori, S. Mrakic-Sposta

**Affiliations:** 1 Department of Health Sciences, Università degli Studi di Milano; 2 Institute of Clinical Physiology, National Research Council (CNR), Milano, Italy

## Abstract

**Introduction:**

Functional Movement Disorders (FMD) are characterized by the presence of neurological symptoms that cannot be explained by typical neurological diseases or other medical conditions. First evidence showed that, compared to healthy controls (CTR), FMD patients presented increased levels of glutamate+glutamine in the anterior cingulate cortex/medial prefrontal cortex, and decreased levels of glutamate in the cerebrospinal fluid, suggesting that a glutamatergic dysfunction might play a role in FMD pathophysiology.

**Objectives:**

According to the evidence of these abnormalities in many neuropsychiatric disorders at level of brain network activity, connectivity, and specific anatomic areas of altered metabolic, and given the evidence of a potential role of glutamate and BDNF in the pathophysiology of FND, in this study we aimed to assess circulating levels of glutamate, BDNF, dopamine, oxidative stress biomarkers, creatinine, neopterin and uric acid in patients with FMD and in a control group of healthy subjects.

**Methods:**

12 FMD patients (4 males, 8 females) and 20 CTR (4 males, 16 females) were recruited and underwent venous blood sampling and urine collection: levels of glutamate, BDNF, dopamine, oxidative stress, creatine, neopterin, and uric acid were analysed. Participants also underwent a psychometric assessment investigating depression, anxiety, and alexithymia.

**Results:**

Levels of glutamate, BDNF and dopamine were significantly lower in the blood of FMD patients than CTR. Glutamate and dopamine levels were positively associated with levels of alexithymia.

**Conclusions:**

Our findings give further evidence that glutamatergic dysfunction might be involved in the pathophysiology of FMD, possibly representing a biomarker of disease; moreover, since glutamatergic and dopaminergic system are closely interconnected, our results might have a relevance in terms of treatment options for FMD patients.

**Disclosure of Interest:**

None Declared

